# Supplementary material for: Emergent patterns of collective cell migration under tubular confinement
Source: Nat Commun. 2017 Nov 15;8:1517. doi: 10.1038/s41467-017-01390-x (PMC5688140; doi:10.1038/s41467-017-01390-x)
Supplement: Supplementary file 1 — Supplementary Information [file 41467_2017_1390_MOESM1_ESM.pdf]

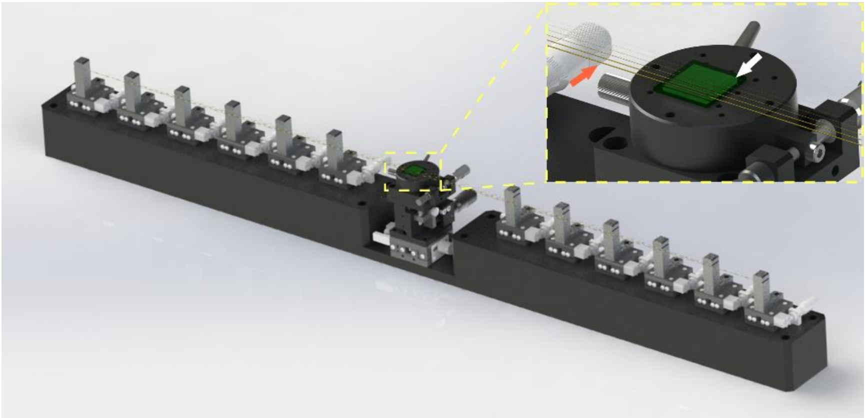

Supplementary Figure 1. The schematic illustration of the precise stage for the alignment of metal wire templates for PDMS microtube fabrication. The inset shows that the metal wires (orange arrow) are alinged in parallel just 1 – 2 μm above a silicon wafer (green slice, white arrow) and pre-cured PDMS is used to replicate the shapes.

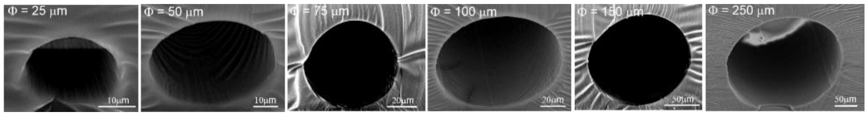

Supplementary Figure 2. Representative SEM images of the microtube openings (with diameters of 25 µm, 50 µm, 75 µm, 100 µm, 150 µm and 250 µm (from left to right)).

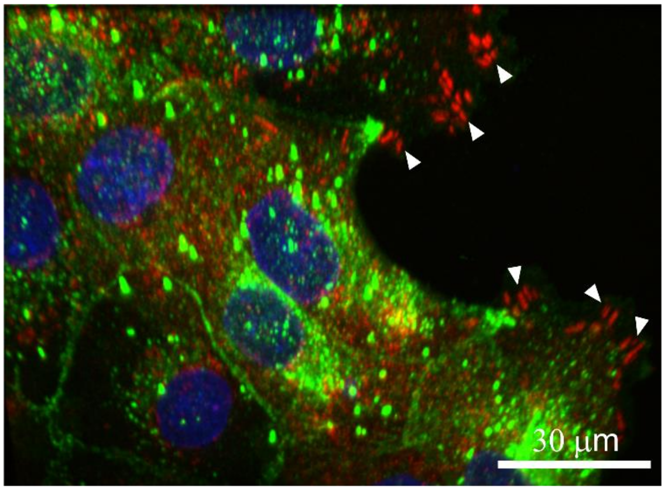

Blue: Nuclei Green: E-cadherin Red:Paxillin

Supplementary Figure 3. Fluorescent image of section of MDCK TCS (nuclei in blue, E-cadherin in green and paxillin in red) formed inside a microtube of 100 µm in diameter. Paxillin staining identifies the focal adhesions formed by cells in the inner surface of the microtube.

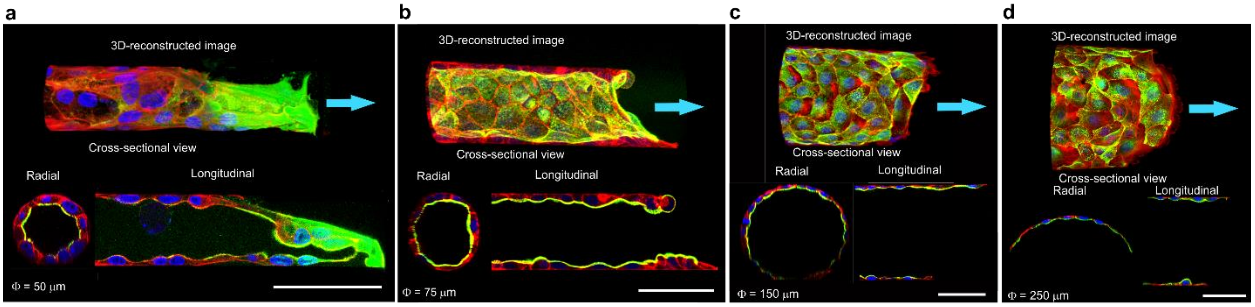

Supplementary Figure 4. 3D reconstructed fluorescent images of anti-Gp135 and phalloidin (red) stained MDCK TCSs (nuclei in blue, DAPI) in microtubes of different diameters: (**a**) 50 µm, (**b**) 75 µm, (**c**) 150 µm and (**d**) 250µm. Lower panels show radial (left) and longitudinal (right) cross-sections of the representative TCSs. Cyan arrows indicate the direction of collective migration. Scale bars: 75 µm.

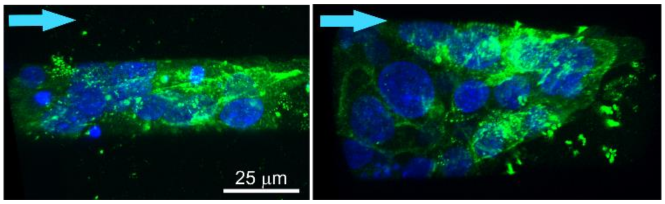

Supplementary Figure 5. 3D reconstructed fluorescent images of MDCK cells (stained for E-cadherin (green) and nuclei (blue)) at the tip of small tubes (left: 25 µm and right: 50 µm). Cyan arrows indicate the direction of collective migration.

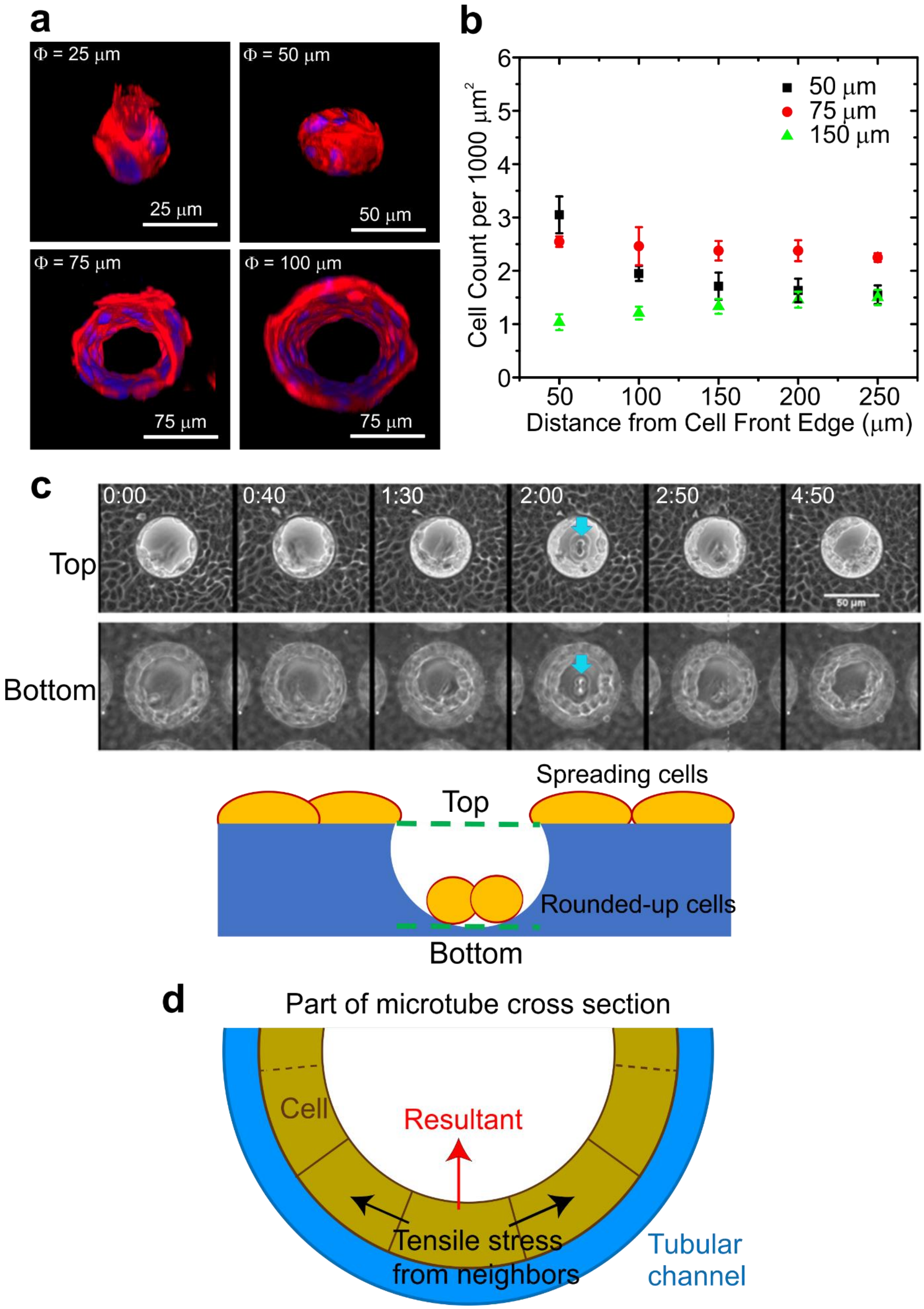

a
Φ = 25 μm
25 μm
Φ = 50 μm
50 μm
Φ = 75 μm
75 μm
Φ = 100 μm
75 μm
b
Cell Count per 1000 μm²
Distance from Cell Front Edge (μm)
50 μm
75 μm
150 μm
c
0:00
0:40
1:30
2:00
2:50
4:50
Top
Bottom
50 μm
Spreading cells
Top
Rounded-up cells
Bottom
d
Part of microtube cross section
Cell
Resultant
Tensile stress
from neighbors
Tubular
channel

Supplementary Figure 6. (**a**) 3D reconstructed fluorescent images of MDCK cells (stained for actin (red) and nuclei (blue)) at the tip of microtubes of different diameters. (**b**) Cell density at different distances from the front edge of the TCSs in various microtubes ($n = 5$ from 3 independent experiments). Data are presented as mean ± s.e.m. (**c**) (top) Phase contrast movies of the tissue spreading condition on flat surfaces and in a 50 μm diameter bowl-shaped structure with negative curvature, with imaging plane at the top and bottom plane of the bowl. Tissue spread well on flat surface at the top plane, while a few cells in the bowl spread transiently for the first three frames, then round up (cyan arrows) in the fourth frame, before migrating out of the bowl structure. Time stamp shows hr:min, scale bar: 50 μm. (bottom) Schematic of the experiment. (**d**) Schematic showing radial force (red arrow) as a resultant of tensile stress (black arrows) from neighbouring cells stretching cell height.

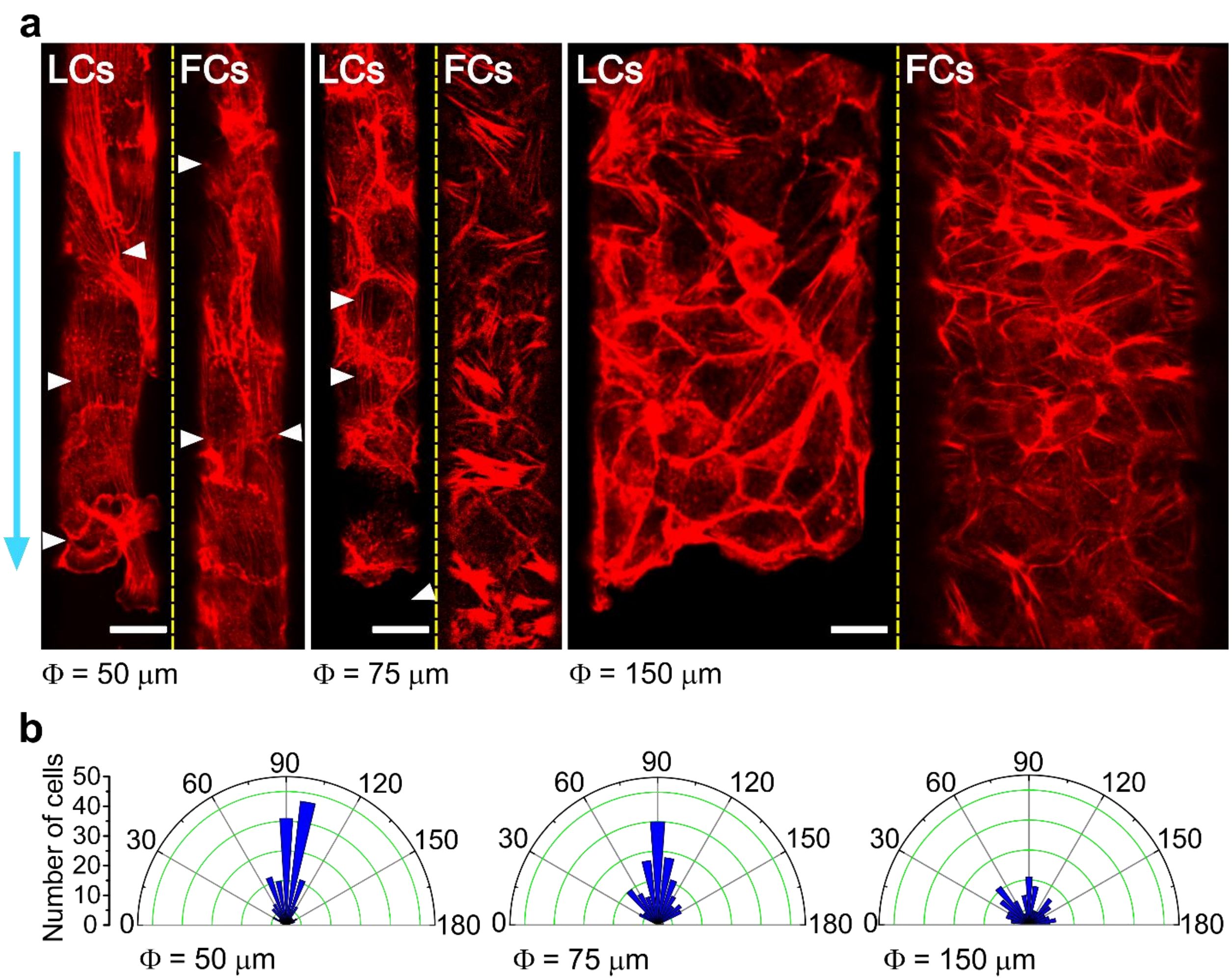

Supplementary Figure 7. (**a**) Fluorescent images of basal actin stress fibers in MDCK WT TCSs of different microtubes. LCs: the leading cell front and FCs: the follower cells. Cyan arrow indicates the direction of collective migration. Scale bars: 20 µm. (**b**) Polar graph plotting the histogram of the cell orientation distribution inside microtubes (50 µm, 75 µm and 150 µm (from left to right)). 90° means oriented along the microtube long-axis and 0° and 180° mean the cell is oriented perpendicular to the long-axis.

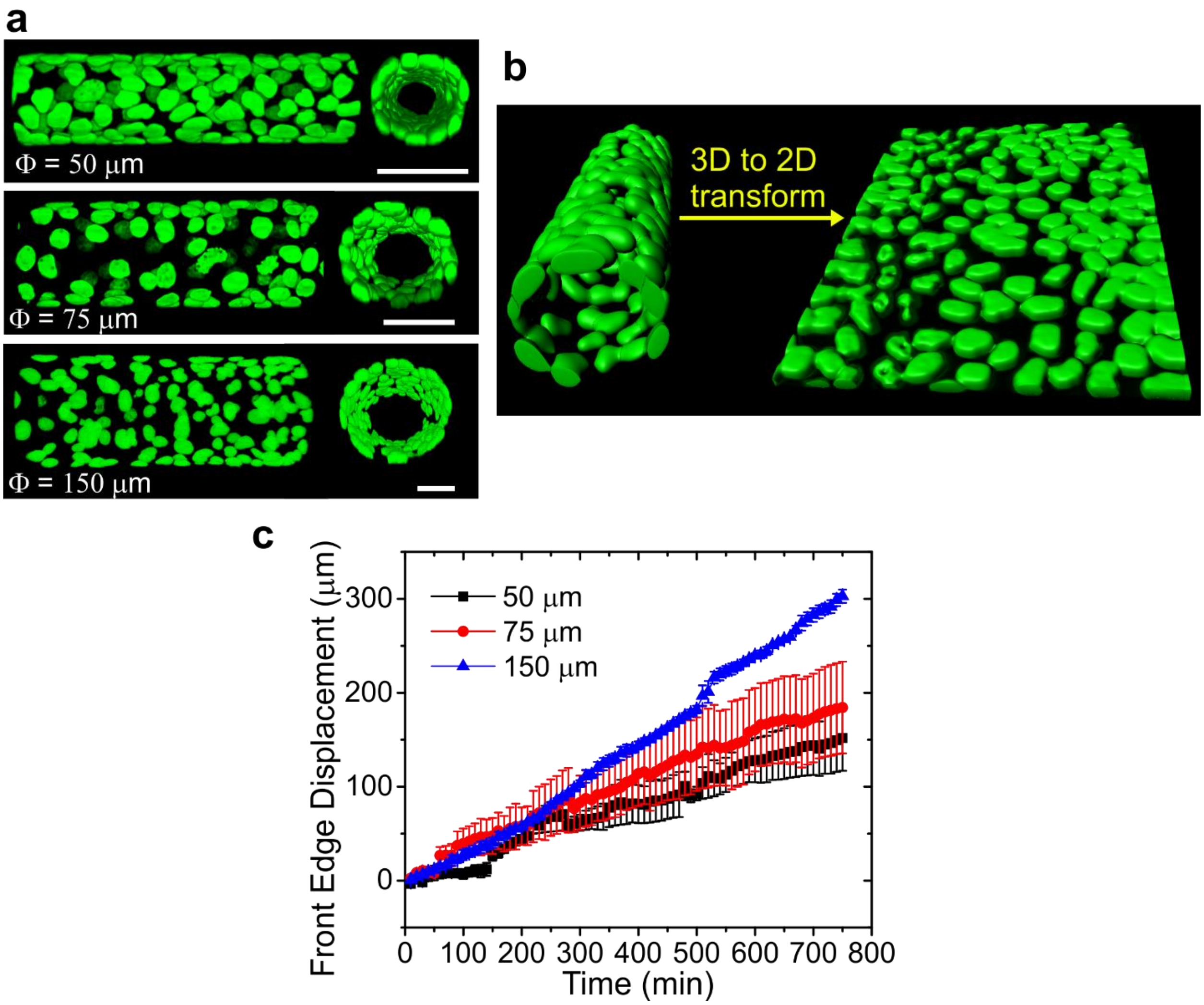

Supplementary Figure 8. (**a**) 3D fluorescent reconstruction of the stably transfected H1-GFP MDCK TCSs in different microtubes showing cell nucleus. Each panel includes the diameter of the microtube (left corner), and the top (left column) and cross-sectional (right column) views of the TCSs. Scale bars: 50 µm. (**b**) Schematic representation of the transformation of 3D tubular cell sheets into 2D equivalent for analysis. Green – nucleus of H1-GFP cells. (**c**) Displacement of MDCK cell fronts in different microtubes (50 µm, 75 µm and 150 µm) as a function of time (n = 3 from 3 independent experiments in each condition). Data are presented as mean ± s.e.m.

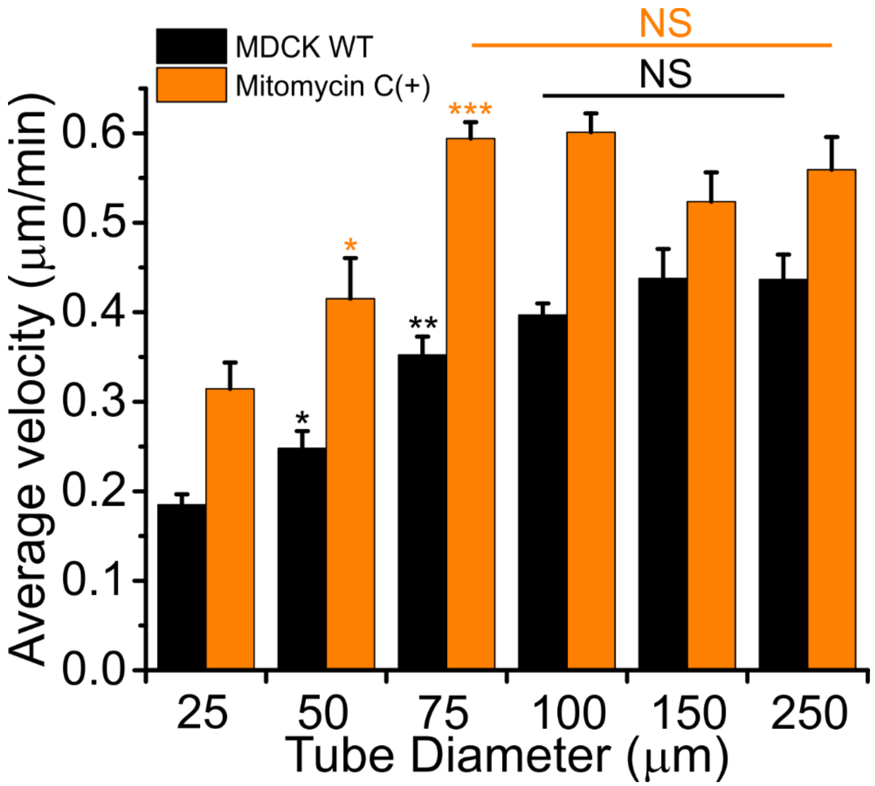

Supplementary Figure 9. Average velocity of tissue front for WT MDCKs and mitomycin C treated WT MDCKs in tubes of different diameters ($n = 8$ from 4 independent experiments in each condition). For each condition, *t*-test between each microtube diameter and 25 μm, unless otherwise indicated by lines, ** $P < 0.01$, *** $P < 0.001$, NS non-significant. The plots represent the mean ± s.e.m.

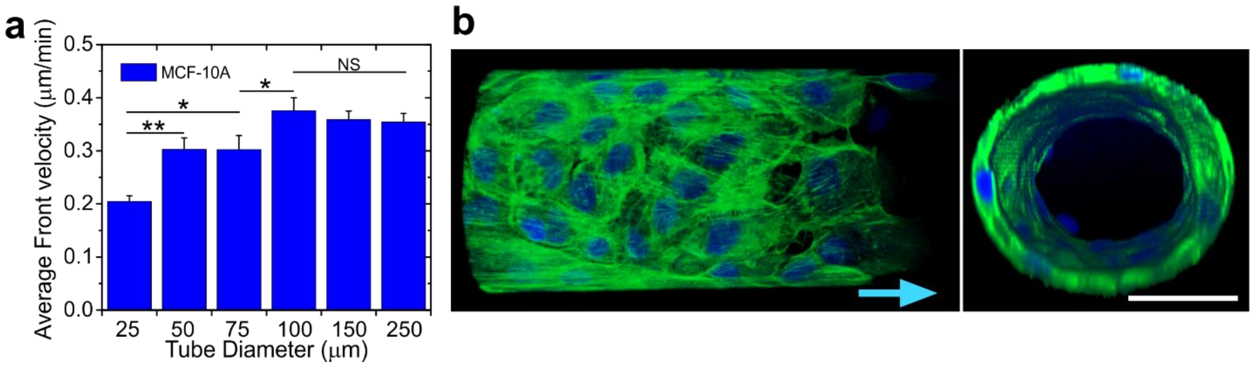

Supplementary Figure 10. (**a**) Average velocity of tissue front for MCF-10A cells in tubes of different diameters (*n* = 6 from 3 independent experiments per condition). (**b**) 3D fluorescent reconstructed image of MCF-10A forming proper tubular structures in 100 μm diameter microtube. DAPI – blue, Phalloidin – green, scale bar: 50 μm. Cyan arrow indicates the direction of collective migration. *t*-test, * $P < 0.05$, ** $P < 0.01$, NS non-significant. The plots represent the mean ± s.e.m.

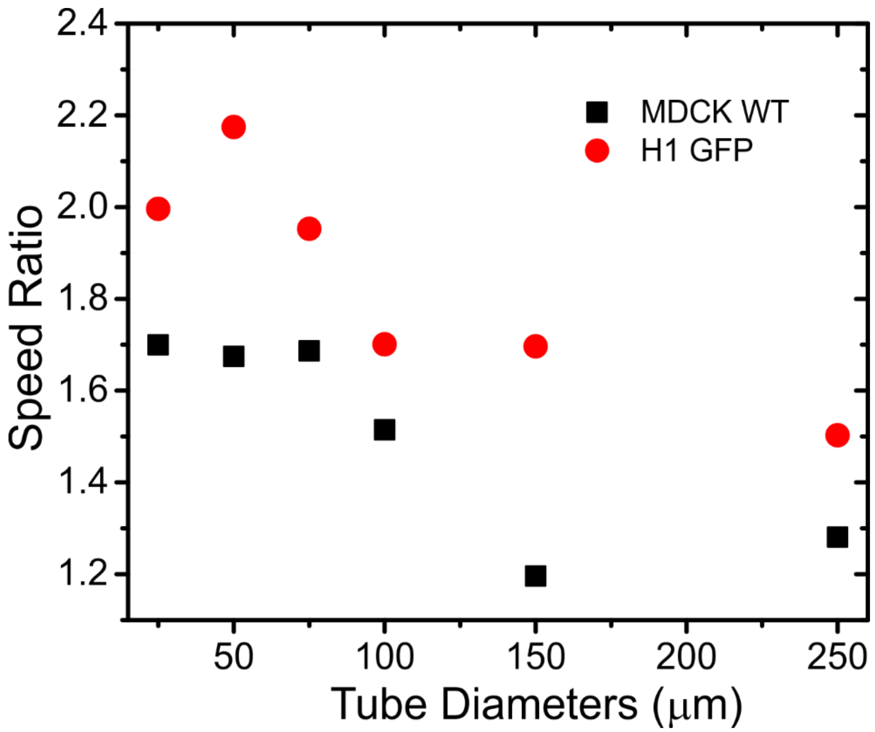

Supplementary Figure 11. Speed-ratio = $\overline{v_f}$ (without proliferation) / $\overline{v_f}$ (with proliferation) for WT MDCKs and H1-GFP MDCKs in various conditions.

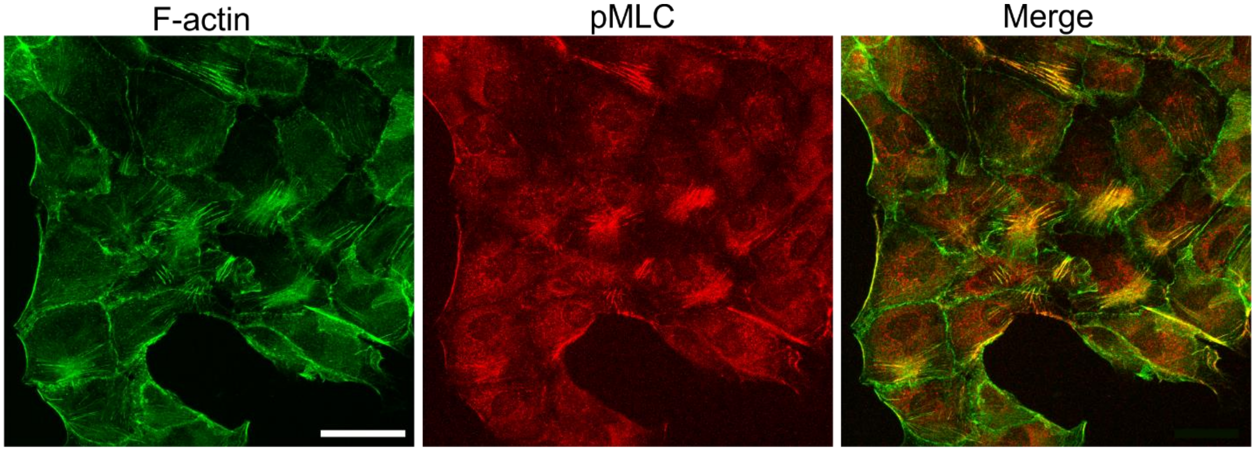

Supplementary Figure 12. MDCK WT cells on flat substrate fixed and stained for actin (green and pMLC (red). Scale bar, 30 μm.
