# Supplementary material for: Emergent patterns of collective cell migration under tubular confinement
Source: Nat Commun. 2017 Nov 15;8:1517. doi: 10.1038/s41467-017-01390-x (PMC5688140; doi:10.1038/s41467-017-01390-x)
Supplement: Supplementary file 3 — Description of Additional Supplementary Files [file 41467_2017_1390_MOESM3_ESM.pdf]

Description of Additional Supplementary Files

File Name: Supplementary Movie 1
Description: MDCK E-cadherin-GFP cells collectively migrating into microtubes of 25 and 100 µm in diameter. Scale bars 20 µm.

File Name: Supplementary Movie 2
Description: MDCK H1-GFP cells collectively migrating into a 100 µm diameter microtube. Scale bar 100 µm.

File Name: Supplementary Movie 3
Description: A 3D movie showing MDCK H1-GFP cells collectively migrating into a 100 µm diameter microtube.

File Name: Supplementary Movie 4
Description: Direction of the velocity fields in H1-GFP MDCK TCS migrating in a microtube of 25 µm in diameter. Each frame is 10 minutes apart and video is played at 10 frames per second.

File Name: Supplementary Movie 5
Description: Direction of the velocity fields in H1-GFP MDCK TCS migrating in a microtube of 100 µm in diameter. Each frame is 10 minutes apart and video is played at 10 frames per second.

File Name: Supplementary Movie 6
Description: Direction of the velocity fields in H1-GFP MDCK TCS migrating in a microtube of 250 µm in diameter. Each frame is 10 minutes apart and video is played at 10 frames per second.

File Name: Supplementary Movie 7
Description: MDCK α-catenin KD cells collectively migrating into a microtube of 100 µm in diameter.

File Name: Supplementary Movie 8
Description: MDCK cells overexpressing Snail transcription factor migrating into a 25 µm diameter microtube.
